# Maternal uniparental disomy of chromosome 7 underlying argininosuccinic aciduria and Silver-Russell syndrome

**DOI:** 10.1038/s41439-022-00211-y

**Published:** 2022-09-12

**Authors:** Atsushi Hattori, Torayuki Okuyama, Tetsumin So, Motomichi Kosuga, Keiko Ichimoto, Kei Murayama, Masayo Kagami, Maki Fukami, Yasuyuki Fukuhara

**Affiliations:** 1 Department of Molecular Endocrinology, National Research Institute for Child Health and Development, Tokyo, 157-8535 Japan; 2 Division of Medical Genetics, National Center for Child Health and Development, Tokyo, 157-8535 Japan; 3 Department of Pediatrics and Clinical Genomics, Saitama Medical University, Saitama, 350-0495 Japan; 4 Center for Medical Genetics, Department of Metabolism, Chiba Children’s Hospital, Chiba, 266-0007 Japan

**Keywords:** Genetics research, Medical genetics, Mutation, Imprinting

## Abstract

We describe a patient presenting with argininosuccinic aciduria and Silver-Russell syndrome (SRS). SRS was caused by maternal uniparental disomy of chromosome 7 (UPD(7)mat). UPD(7)mat also unmasked a maternally inherited splicing variant in *ASL* on chromosome 7, leading to the onset of argininosuccinic aciduria. The phenotype of the present case was more severe than that of a previous case, demonstrating a phenotypic variation in the combination of argininosuccinic aciduria and SRS.

Uniparental disomy (UPD) is a condition in which both copies of a chromosome pair are inherited from one parent^1^. Heterodisomy results from inheriting two different alleles from one parent, whereas isodisomy results from inheriting two copies of a single allele. Both heterodisomy and isodisomy can cause imprinting disorders, whereas isodisomy can unmask recessive disorders^2,3^. Occasionally, isodisomy causes both imprinting disorders and recessive disorders in one patient^4–7^.

Argininosuccinic aciduria (ASA) is the second most common urea cycle disorder^8^. ASA is an autosomal recessive disorder caused by biallelic pathogenic variants in *ASL* (MIM #608310) on chromosome 7. Patients with ASA can present with a neonatal-onset hyperammonemic crisis or a broad late-onset phenotypic spectrum ranging from hyperammonemic crisis to slowly progressive neurocognitive signs and symptoms without apparent hyperammonemia^8^.

Silver-Russell syndrome (SRS) is characterized by prenatal and postnatal growth retardation, relative macrocephaly, body asymmetry, feeding difficulty, and a prominent forehead^9^. Additional clinical features include triangular face, fifth finger clinodactyly and brachydactyly, scoliosis, excessive sweating, and hypoglycemia. SRS is a genetically heterogeneous syndrome; maternal UPD of chromosome 7 (UPD(7)mat) has been identified in 5–10% of patients with SRS^9,10^.

Previously, Li et al.^11^ described a girl with ASA and SRS. The patient had a pathogenic variant of *ASL* (NM_000048.4:c.2 T > A) unmasked by maternal isodisomy of chromosome 7. Her phenotype was relatively mild compared to that of patients with neonatal-onset ASA. As no further cases have been reported, it remains unknown whether patients with ASA and SRS show phenotypic variations. Here, we report another patient with this combination. The phenotype of the present case was more severe than that of the previous case.

The patient in the present report was a boy who was the second child of nonconsanguineous Japanese parents without any family history of congenital disorders. Fetal growth restriction was noted during pregnancy. He was born at 38 weeks of gestation without complications during delivery. He was small for gestational age and had relative macrocephaly (Supplementary Table S1). Recurrent vomiting and wheezing started three days after birth, and he was admitted to the NICU of a community hospital. His respiratory condition worsened, prompting mechanical ventilation starting on the fifth day of life. At the age of 10 days, he was noted to have severe hyperammonemia (526 μmol/L; reference range, 25–85) (Supplementary Table S1). Plasma amino-acid analysis showed elevated levels of citrulline (423.6 nmol/mL; reference range, 20.4–44.8) and argininosuccinic acid (42.0 μmol/L; reference range, <1.5), which was consistent with ASA. He recovered from the crisis by continuous hemodiafiltration and achieved good control of blood ammonium levels (~25 μmol/L) after the initiation of long-term therapy, including tube feeding, protein restriction (1.3–1.5 g/kg/day), arginine supplementation (0.1–0.2 g/kg/day), and the administration of nitrogen-scavenging agents (sodium phenylbutyrate, 0.1–0.2 g/kg/day; sodium benzoate, 0.1–0.2 g/kg/day). However, recurrent vomiting persisted. Severe growth retardation was also noted (Fig. 1A), which prompted gastrostomy at 2 years. The gastrostomy, however, did not significantly improve his nutritional condition because vomiting persisted. He also had neuropsychomotor developmental delay (Supplementary Tables S1 and S2). Head MRI examinations at 1 month and 2 years of age revealed no abnormal findings.

**Fig. 1:**
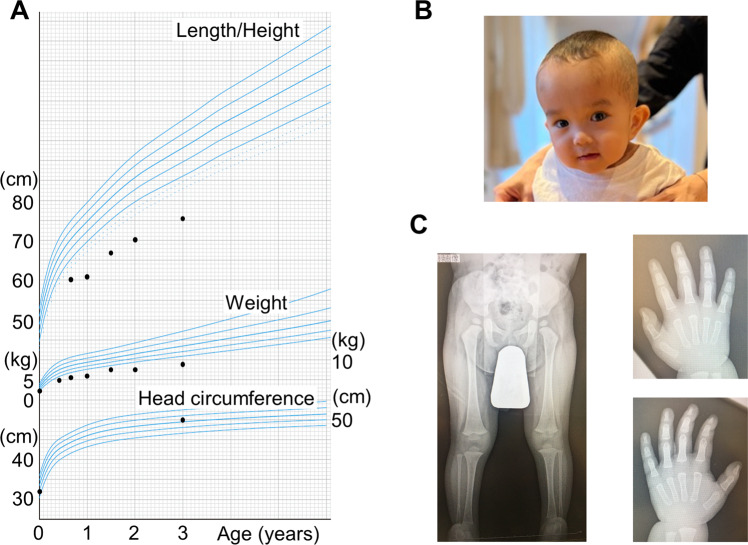
Clinical findings of the patient. **A** Growth chart. The chart was downloaded from the website of the Japanese Society for Pediatric Endocrinology (http://jspe.umin.jp/medical/files_chart/CGC2_boy0-6_eng.pdf). Five curves for each parameter represent +2SD, +1SD, 0SD, −1SD, and −2SD from the mean. **B** Photograph. The patient had relative macrocephaly and brittle and short brush-like hair. **C** Radiographic findings. An X-ray of the lower extremities (left panel) shows mild asymmetry of the lengths of the femurs and tibias. X-rays of the hands (right panels) show the fifth finger brachydactyly.

The patient had several SRS-like signs. His remarkable prenatal and postnatal growth retardation and severe feeding difficulty were compatible with SRS. He also had body asymmetry and relative macrocephaly with a prominent forehead (Fig. 1A–C). His clinical features fulfilled all six diagnostic criteria of SRS (Netchine-Harbison clinical scoring system; meeting four or more criteria confirms the diagnosis)^9^. He also had brachydactyly of the fifth fingers (Fig. 1C) and excessive perspiration. No episode of hypoglycemia was recognized.

Sequencing analysis with a panel including causative genes for urea cycle disorders (*ARG1*, *ASL*, *ASS1*, *CPS1*, *NAGS*, *OTC*, and *SLC25A15*) revealed a homozygous variant in intron 15 of *ASL* (NC_000007:c.1144-9 G > A) in the patient. To clarify whether the variant caused splicing alterations, we sequenced *ASL* transcripts in immortalized lymphoblastoid cell lines established from the peripheral lymphocytes of the patient and his parents. We amplified a region encompassing the boundary of exons 15 and 16 of *ASL* (primer sequences: forward primer, 5′-CACCAAGAGAACATGGGACA-3′; reverse primer, 5′-CCTGCAGTGACAGCTGGTT-3′) to perform direct sequencing. As a result, we observed a splicing alteration that resulted in a seven-nucleotide insertion (c.1143_1144insCACCCAG) in the transcripts of the patient and his mother, whereas the splicing in the father was normal (Fig. 2A, B). The insertion observed in the patient and his mother was predicted to result in the p.(Met382Hisfs*94) variant with the stop codon in the last exon. The variant was predicted to disrupt a C-terminal region involved in four enzymatically active sites of the homotetramers of the ASL protein (Supplementary Fig. S1), leading to a loss of function of the enzyme. The same variant was previously identified in a patient with ASA^12^.

**Fig. 2:**
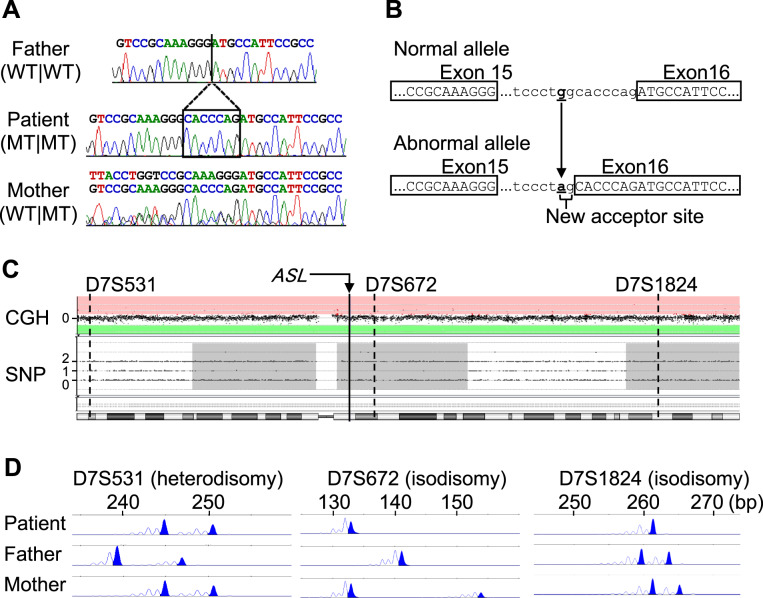
Results of genetic analysis for the patient and his parents. **A** Results of RT‒PCR sequencing. The vertical line in the top panel describes the boundary between exons 15 and 16 of the normal *ASL* transcript. The box highlights a seven-nucleotide insertion. All chromatograms show the data sequenced with the reverse primer. WT wild type, MT mutant. **B** Schema of the intron‒exon boundaries of the normal and abnormal alleles. Exon sequences are written in upper case, whereas intron sequences are written in lower case. The underlined letters describe nucleotide substitutions. **C** Microarray-based comparative genomic hybridization (CGH) and SNP genotyping of chromosome 7. The SurePrint G3 ISCA CGH + SNP Microarray Kit, 4 × 180 K (Agilent Technologies, Santa Clara, CA, USA) was used. For CGH data, the value 0 represents the normal copy number. Log_2_ ratios ≥0.58 (pink area) indicate amplification, whereas values ≤−1.0 (green area) indicate deletion. For SNP genotyping data, values of 0 and 2 correspond to homozygous genotypes, while a value of 1 corresponds to heterozygous genotypes. Gray areas describe genomic regions that have lost heterozygosity. The solid vertical line describes the position of *ASL*, and the dashed vertical lines describe the positions of the markers evaluated by microsatellite analysis. **D** Representative results of microsatellite analysis for the patient and his parents. The x-axis describes the sizes of the PCR products.

Trio genotyping showed that the patient’s mother had the same variant in a heterozygous state, whereas the father did not have the variant. These results indicated that the patient had UPD(7)mat or hemizygosity associated with paternal deletion. To distinguish these two conditions, we performed microarray-based comparative genomic hybridization and SNP genotyping (Fig. 2C). As a result, we found that the patient had loss of heterozygosity (LOH) on two segmental regions (~93 Mb in total) of chromosome 7, one of which encompassed *ASL* (Fig. 2C). No other chromosome had an LOH of >5 Mb. No copy number alteration was identified in chromosome 7. These results strongly suggested that this patient had a maternal isodisomy encompassing *ASL*. Moreover, microsatellite analysis for nine loci on chromosome 7 (Supplementary Table S3) showed that the patient had maternal UPD of chromosome 7, which consisted of both heterodisomy and isodisomy (Fig. 2D and Supplementary Table S3).

This report describes a patient who presented with the comorbidity of ASA and SRS. ASA was presumably caused by a pathogenic intronic variant of *ASL* in the patient, which was unmasked by maternal isodisomy. The intronic variant resulted in a splicing alteration that caused a translational frameshift. The abnormal transcript probably escaped nonsense-mediated mRNA decay, given the frameshift with the premature termination codon in the last exon^13^. Nevertheless, this variant was likely pathogenic because the affected C-terminal region was involved in the four enzymatic sites of the ASL homotetramer (Supplementary Fig. S1)^14^. The SRS-like phenotypes of the patient (Supplementary Table S1) cannot be explained by ASA. Along with these SRS-like signs, atypical trio genotypes (homozygous variants in the child with the absence of the same variant in the father) strongly indicated that UPD(7)mat contributed to the phenotype. Microsatellite analysis confirmed UPD(7)mat.

Thus far, only one case report describes ASA caused by an *ASL* variant unmasked by UPD(7)mat^11^. Overall, the phenotype of the previously reported patient was much milder than that of our patient (Supplementary Table S1). There are two possible explanations for these phenotypic differences. First, the *ASL* variant in the present case is predicted to be more devastating than that in the previous case. Zielonka et al.^15^ suggested that variant-dependent ASL enzymatic activity determines clinical severity; ≤9% of enzymatic activity has been linked to a severe phenotype. The c.1144-9 G > A variant in the present case was expected to alter C-terminal amino acids, which include the main components of the enzymatic sites in the ASL tetramer (Supplementary Fig. S1). Indeed, the variant affects the arginine residue at the 385th position, whose alteration causes complete loss of ASL enzymatic activity^15^. In contrast, the variant in the previous case (c.2 T > A) was predicted to delete 20 amino acids at the N-terminus (p.Ala2_Met21del). The only known pathogenic variant in the affected region (c.35 G > A, p.(Arg12Gln)) retained 15% enzymatic activity^15^. Second, the severe feeding difficulty in the present cause, probably due to SRS, might have accelerated the neurotoxic effect of ASA. Generally, insufficient energy intake promotes catabolism and predisposes patients with urea cycle disorders to hyperammonemic crisis^16^. The patient in the present report had recurrent vomiting even after the placement of a gastrostomy tube, whereas the previously reported patient only had intermittent vomiting without requiring tube feeding.

Isodisomy is an important etiology underlying autosomal or X-linked recessive disorders. A previous report demonstrated that one out of 2000 individuals from a general population had UPD of one or two chromosomes^17^. More than half of the identified UPD was complete or partial isodisomy. We expect that there are other cases of ASA combined with SRS due to UPD(7)mat. Considering that feeding difficulty due to SRS theoretically may promote catabolism, the possible coexistence of SRS should be considered during the management of ASA.

In conclusion, we presented a patient in whom UPD(7)mat caused ASA and SRS. The present case report demonstrates the phenotypic variation in cases of ASA combined with SRS and suggests that the possible complications of SRS should be considered in the management of ASA.

## HGV database

The relevant data from this Data Report are hosted at the Human Genome Variation Database at 10.6084/m9.figshare.hgv.3222.

## Supplementary information

Supplementary Material
