# Supplementary material for: Maternal uniparental disomy of chromosome 7 underlying argininosuccinic aciduria and Silver-Russell syndrome
Source: Hum Genome Var. 2022 Sep 12;9:32. doi: 10.1038/s41439-022-00211-y (PMC9468177; doi:10.1038/s41439-022-00211-y)
Supplement: Supplementary file 1 — Supplementary Material [file 41439_2022_211_MOESM1_ESM.docx]

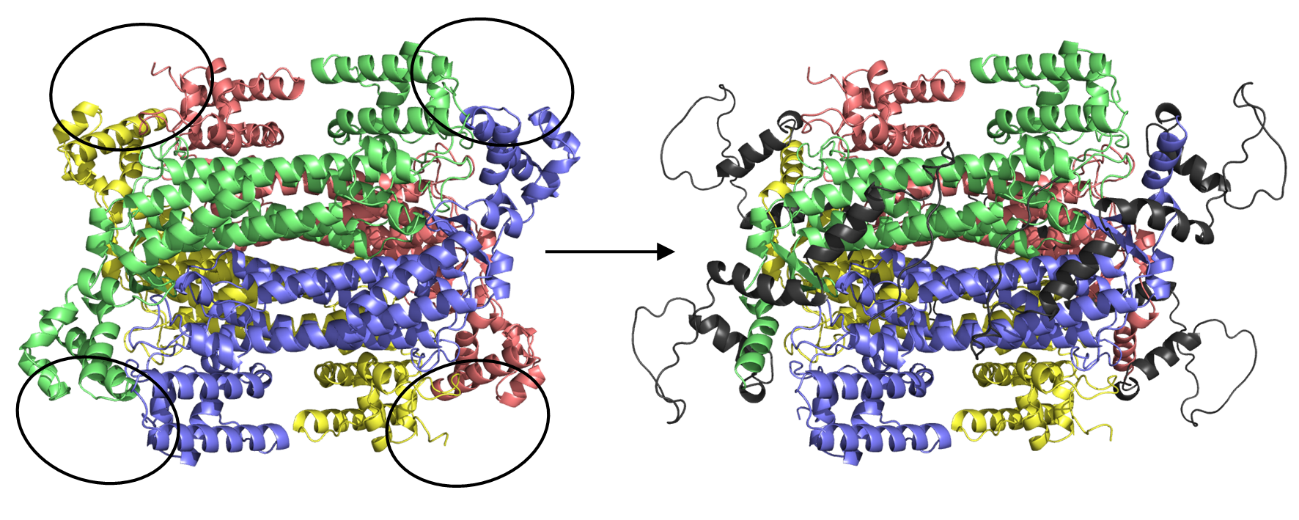


**Supplementary Figure S1. Predicted structure of argininosuccinate lyase (ASL) homotetramer.**

The left panel shows the structural model of wild type ASL tetramer. The four subunits are colored in red, blue, yellow, and green. The four circles highlight the active sites of the tetrameric enzyme. The right panel shows the predicted structure of the mutant ASL identified in the present study (p.Met382Hisfs*94). The black color highlights the C-terminal regions of the four subunits that are altered by the pathogenic variant. The tetrameric structure model was created by the following two steps: (i) predicting the structures of the ASL full-length subunit and the core frame of the tetramer (a complex consisting of four identical fragments (p.Asp212–p.Leu353) of ASL) by AlphaFold2 (https://colab.research.google.com/github/sokrypton/ColabFold/blob/main/ AlphaFold2.ipynb) from the ASL amino acid sequence (P04424) obtained at the UniProt database (https://www.uniprot.org/), (ii) assembling the entire tetramer structure by aligning the four full-length subunits to the core frame of the tetramer using PyMOL.

Supplementary Table S1. Molecular and clinical features of the present case and the previous case

|  | Present case | Li et al., 2014 |
| --- | --- | --- |
| *ASL* variant | c.1144-9G>A  p.(Met382Hisfs*94) | c.2T>A  p.(Ala2_Met21del) |
| At birth | | |
| Gestational age | 38 weeks 0 day | 37 weeks |
| Length | 43.5 cm | NA |
| Weight | 2100 g (−2.45 SD) | 2080 g (<3rd percentile) |
| Head circumference | 32.0 cm (−0.74 SD) | NA |
| At onset | | |
| Age | 10 days | 1 year |
| NH_3_ | 526 μmol/L  (Reference, 25–85) | 320 μmol/L  (Reference, 21–78) |
| During chronic phase | | |
| Age | 3 years | 2 years |
| Height | 75.5 cm (−5.1 SD) | 10th percentile |
| Weight | 8.8 kg (−3.2 SD) | 10th percentile |
| Developmental delay^a^ | Achieved walking at 3 years old  Could not speak at 3 years old | No developmental delay was described |
| Netchine-Harbison clinical scoring system | | |
| Total score | 6/6 | 1–4/6 |
| Birth length and/or weight  ≤ −2.0 SD | Yes | NA |
| Postnatal growth failure  (height ≤ −2.0 SD | Yes | No |
| Relative macrocephaly | Yes | NA |
| Prominent forehead | Yes | Yes |
| Body asymmetry | Yes | NA |
| Feeding difficulty and/or  BMI ≤ −2.0 SD | Yes | No |

^a^Details of developmental assessment are described in Supplementary Table S2

BMI, body mass index; NA, not available; SD, standard deviation.

Supplementary Table S2. Developmental evaluation according to the Enjoji Scale

| Assessment categories | Developmental quotient | |
| --- | --- | --- |
|  | 3 years 0 month^a^ | 3 years 7 months^a^ |
| Locomotor activity | 33 | 37 |
| Manual activity (fine motor skills) | 39 | 37 |
| Personal activities of daily living | 28 | 33 |
| Social activities | 39 | 37 |
| Speech | 28 | 23 |
| Language recognition | 33 | 37 |

Developmental quotient was calculated as follows;

$$Developmental quotient= \frac{Developmental age}{Chronological age} \times100$$

Details of the Enjoji Scale were described previously [Enjoji, M. & Yanai, N. Analytic test for development in infancy and childhood. *Pediatr. Int.* **4**, 2–6 (1961); Enjoji M. *Commentary on Enjoji Scale of analytic test for development in infancy and childhood. New edition revised by Department of Pediatrics at Kyushu University*. (Keio University Press, 2009) (in Japanese)].

^a^Age at examination.

Supplementary Table S3. Results of microsatellite analysis

| Locus | Location | PCR product size (bp) | | | Assessment |
| --- | --- | --- | --- | --- | --- |
|  |  | Patient | Father | Mother |  |
| D7S531 | 7p22.2 | 245/251 | 239/247 | 245/251 | Maternal heterodisomy |
| D7S484 | 7p14.2 | 99/99 | 101/109 | 99/101 | Maternal isodisomy |
| D7S2846 | 7p14.1 | 175/175 | 179/187 | 175/183 | Maternal isodisomy |
| D7S519 | 7p12.3 | 261/261 | 259/263 | 261/265 | Maternal isodisomy |
| D7S672 | 7q11.22 | 132/132 | 140/140 | 132/154 | Maternal isodisomy |
| D7S669 | 7q21.11 | 127/127 | 125/127 | 117/127 | Not informative |
| D7S684 | 7q34 | 179/179 | 177/179 | 173/179 | Not informative |

The methods of microsatellite analysis were described previously [Fuke, T. *et al.* Molecular and clinical studies in 138 Japanese patients with Silver-Russell syndrome. *PloS One* **8**, e60105 (2013)].
